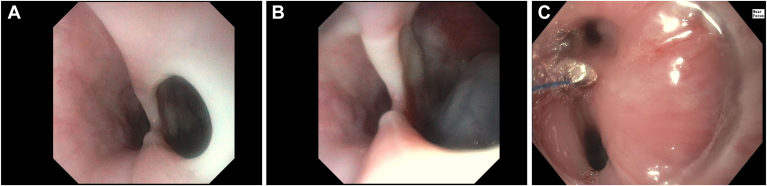# A Tracheoesophageal Fistula Caused by a Tracheal Cuff Balloon

**DOI:** 10.1016/j.gastha.2023.07.006

**Published:** 2023-07-18

**Authors:** Jamie O. Yang, Soonwook Hong, Wendy Ho

**Affiliations:** 1 Department of Internal Medicine, University of California, Los Angeles, Los Angeles, California; 2 Division of Digestive Diseases, University of California, Los Angeles, Los Angeles, California

A 58-year-old previously healthy male developed COVID fibrosis/ acute respiratory distress syndrome requiring tracheostomy and percutaneous endoscopic gastrotomy tube placement. His hospital course was complicated by recurrent aspiration, pneumonias, and tracheomalacia with air leak requiring upsizing of the tracheostomy. He was evaluated and deemed to not be a lung transplant candidate.

Due to bloody gastrotomy tube output, an esophagogastroduodenoscopy was performed, which found a large 2 cm tracheoesophageal fistula just below the upper esophageal sphincter (Figure A), through which the tracheostomy balloon was visualized (Figure B). The tracheoesophageal fistula was thought to be caused by erosion from the balloon, and the likely cause of the patient’s recurrent pneumonias and air leak issues.

About 1 week later, he underwent a second endoscopy to repair the defect. Hemoclips were attempted initially, but the edges of the defect were too fibrotic to allow for effective tissue approximation. The defect was ultimately closed with eight HeliX Tacks and reinforced with a hemoclip (Figure C). A repeat bronchoscopy two days later showed trachea outpouching, but no evidence of fistula, and the tracheostomy cuff was advanced distal to the outpouching. The patient was placed on comfort care and passed from sepsis 6 weeks later.

**Figure:**